# Effects of fetal bovine serum and estrus buffalo serum on maturation of buffalo (*Bubalus bubalis*) oocytes *in vitro*

**DOI:** 10.14202/vetworld.2015.143-146

**Published:** 2015-02-09

**Authors:** Gopal Puri, S. S. Chaudhary, V. K. Singh, A. K. Sharma

**Affiliations:** Department of Veterinary Physiology & Biochemistry, Vanbandhu College of Veterinary Science & Animal Husbandry, Navsari Agricultural University, Navsari - 396 450, Gujarat, India

**Keywords:** buffalo oocytes, estrus buffalo serum, fetal bovine serum, *in vitro* maturation

## Abstract

**Aim:**

The aim was to assess the effects of fetal bovine serum (FBS) and estrus buffalo serum (EBS) on *in vitro* maturation rate of oocytes in buffalo.

**Materials and Methods:**

Maturation rate of oocytes was assessed in two maturation media supplemented with 20% FBS and EBS. Oocytes maturation rate was evaluated on the basis of cumulus cell expansion and extrusion of polar body after 24 h of *in vitro* culture in CO_2_ incubator.

**Results:**

The average percentage of *in vitro* matured oocytes in FBS was 83.80%, and EBS was 77.45%, respectively. The results revealed a significant (p<0.05) increase in maturation rate of oocytes in FBS than EBS.

**Conclusion:**

Buffalo oocytes were better *in vitro* matured in FBS than EBS.

## Introduction

*In vitro* cell or tissue cultures play an important role for research in science and industry. Fetal bovine serum (FBS) is a common supplement to *in vitro* and *ex vivo* cell, tissue and organ cultures [1]. FBS contains essential components hormones, vitamins, transport proteins, and attachment, spreading and growth factors [2]. It is estimated that about 500,000 L of serum are produced on an annual basis, for this purpose more than 1,000,000 bovine fetuses have to be harvested annually for obtaining fetal calf serum [3]. Methods to reduce the requirements for FBS in culture media as well as alternative animal serum substitutions were described earlier [4]. The removal of serum from the cell culture medium or the replacement with other complex biological fluids and extracts initiates multifarious variations in the interactive nature of the cell culture system.

*In vitro* maturation (*IVM*) and *in vitro* fertilization (*IVF)* procedures performed on oocytes obtained from slaughter-house derived ovaries have recently provided a practical means for producing large number of bovine zygotes at low cost for research and commercial settings [5]. The oocytes maturation is a crucial step for the generation of quality oocytes capable of being fertilized and undergoing normal embryonic development into blastocyst after *IVF* [6]. There have been reports to enhance early development of *in vitro* produced embryos in domestic animals by addition of sera, hormones, and somatic cells [7]. Supplementation of serum in media had a biphasic favorable effect on maturation. The serum contains a number of known growth factors that have an important role in the regulation of oocyte maturation, particularly via cumulus cells it also prevents the hardening of the zona pellucida; moreover, the beneficial action of serum may be due to its anti-oxidant properties [8].

The present experiment was designed to assess the effects of fetal bovine serum (FBS) and estrus buffalo serum (EBS) on *IVM* rate of oocytes in buffalo.

## Materials and Methods

### Collection of oocytes

Buffalo ovaries of unknown reproductive status were collected from local slaughter house and carried to the laboratory in normal saline solution (0.85% NaCl) fortified with gentamicin (50 μg/ml) in a thermo flask at 37-38°C within 2 h of slaughter. In the laboratory, extraneous tissue was removed, and ovaries were thoroughly washed with 70% ethanol followed by three rinses in phosphate buffer saline solution.

### Grading of oocytes

Oocytes were aspirated from all the visible non-atretic surface follicles of the ovary by using 5 ml sterile syringe fitted with 18 G needle containing oocytes collection medium after final washing. The searching of oocytes was carried out in oocytes collection media under stereo zoom microscope. Cumulus oocytes complexes (COCs) were recovered by scoring method [9]. Oocytes possessing a full cumulus mass, unfragmented cytoplasm, and intact zona were selected for further processing. The COCs were evaluated and graded by the methods [10]. Good and excellent quality oocytes having more than 3-5 cumulus cell layers were cultured in 50 μl droplets (20-25 oocytes/droplet) of maturation media in 35 mm sterile petri dish.

### IVM of oocytes

The excellent (>5 layers) and good (>3 layers) quality of COCs were selected for *IVM*. The selected COCs were divided randomly into two groups for maturation in culture: (i) *IVM* medium (TCM-199 with HEPES media, 10% buffalo follicular fluid, 0.5 μg/ml follicle-stimulating hormone, 10 μg/ml luteinizing hormone, 0.5 μg/ml estradiol) supplemented with 20% FBS (FBS; Sigma, USA); (ii) *IVM* medium supplemented with 20% EBS obtained from estrus buffalo. Selected COCs were cultured in sterile tissue culture petri dish (35 mm×35 mm; Axiva) containing 100 µl of *IVM* medium at 38.5°C in an atmosphere of 5% CO_2_ and 95% air with high humidity in CO_2_ incubator for 24 h. Following 24 h of culture, matured oocytes in both the drops were observed on the basis of cumulus cell expansion, extrusion of first polar body and metaphase II plate were indicators of oocyte maturation and considered as matured oocytes.

## Results

**Figure-1:**
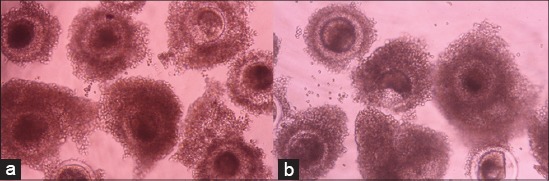
*In vitro* maturation of buffalo oocytes in fetal bovine serum (FBS) and estrus buffalo serum (EBS) supplemented maturation media, (a) *In vitro* maturation of oocytes in FBS (×20), (b) *in vitro* maturation of oocytes in EBS (×20).

The effect of supplementation of 20% FBS and EBS in maturation media on the maturation rate of buffalo oocytes is given in Table-1 and Figure-1. The results revealed a significant (p<0.05) increase in maturation rate when the maturation media was supplemented with FBS than in EBS supplemented media. The percentage of *IVM* of oocytes in TCM-199 with supplementation of 20% FBS was significantly higher (83.80%) than in EBS (77.45%). *IVM* of buffalo oocytes was significantly (p<0.05) better in FBS as compared to EBS, which might be due to the effect of growth factors, nutrients and anti-oxidant present in FBS. Thus, FBS as a supplement in maturation media increases the *IVM* rate of bovine oocytes better than EBS.

**Table-1:** *In vitro* maturation rate of buffalo oocytes in FBS and EBS supplemented maturation medium.

Total number of ovary used	Oocyte/ovary	Culturable oocytes	Number of oocytes cultured (20%)		Matured oocytes (%)	
	
			FBS	EBS	FBS	EBS
266	1.16	207	105	102	88 (83.80)	79 (77.45)

FBS=Fetal bovine serum, EBS=Estrus buffalo serum

## Discussion

Immature bovine oocytes cultured in standard maturation medium, they resume the first meiotic division which is essential for oocytes to achieve full developmental competence for fertilization. The alteration of basic maturation conditions can affect oocyte competence significantly as reflected by the morula and blastocyst yield after *IVF* [11]. A number of ultrastructural and molecular changes occurring during oocytes development are linked to the developmental competence of the gamete [12].

In most studies on *IVM* of animal oocytes, the basic medium is supplemented with different kinds of sera [13]. Serum is commonly used as a supplement to cell culture media [14]. FBS has been considered as a universal growth media for cell culture. It can be used as culture additive for the stimulation of cellular proliferation, and it contains essential components such as hormones, vitamins, transport proteins and attachment, spreading and growth factors [2]. The serum contains a number of known growth factors that have an important role in the regulation of oocyte maturation, particularly via cumulus cells it also prevents the hardening of the zona pellucida. Moreover, beneficial action of serum may be due to its anti-oxidant properties [8]. The major functions of serum are to provide hormonal factors which stimulate cell growth and proliferation, promotes differentiated functions, transport proteins carrying hormones, minerals trace elements, lipids, attachment and spreading factors and stabilizing and detoxifying factors [14]. A favorable effect of estrus cow serum when added to the maturation medium promotes the rupture of germinal vesicle and induces oocytes maturation [15]. Basic media supplemented with, estrus sheep serum and estrus goat serum supported better rates of *IVM, IVF* and embryo development of ovine oocyte [16]. Hence, supplementation of media with serum had a biphasic favorable effect on maturation. The beneficial effects of serum for oocyte maturation may also be via cumulus cells or directly on the oocytes. Supplementation of buffalo follicular fluid and FBS at different fraction obtained by ultracentrifugation, are effective maturation medium, as they promoted the development of high quality matured bovine oocytes [17]. FBS can be supplemented in *IVM* and *IVF* media to achieve better maturation and cleavage [18]. Oocytes for *IVM* are typically selected supported their cumulus investment and homogeneity of cytoplasm. The gap junctions between oocyte and cumulus cells were needed for the transport of growth factors and hormones necessary for normal maturation and developmental competence [19,20]. Various protein supplements are essential for cellular modifications (specialization of cell) and growth in various living systems that support the interactions with cellular proliferation and MAPK networks [21]. The addition of different thiol compounds (cystine, cysteine, cysteamine, glutathione [GSH], β mercaepthanol) to the *IVM* media improve embryo development, increases intra-cytoplasmic GSH concentration and protects cells from culture oxidative stress [22,23]. The effect of supplementation of synthetic oviduct fluid with glucose and vitamins has been experimented [24] during the first step of the *in vitro* culture of ovine zygotes. Addition of hydrolyzed plant proteins have anti-apoptotic properties [25] and contains numerous free amino-acids, short oligopeptides, some vitamins and carbohydrates, which may cumulatively be responsible for improved embryo production [26]. Supplementation of protein additives informs ovine amniotic fluid, FBS or sheep serum to culture media are more efficacious for *IVM* of sheep oocytes [27]. In contrary, a better cleavage rate was achieved with the static culture method supplemented with EBS, as compared to static methods with FCS and flux culture methods with either EBS or FCS in buffalo and *IVM* of oocyte with static culture containing EBS would be a potential method to reduce the cost of laboratory produced buffalo embryos [28]. Addition of hormone combinations with FBS to culture media could significantly improve the *IVM* of sheep oocytes [29]. In the present study, *IVM* rate of buffalo oocytes was enhanced in FBS supplemented maturation medium while in EBS, maturation rate was slower.

## Conclusion

The maturation rate of oocytes was higher in FBS than EBS, which might be due to the effect of growth factors, nutrients and anti-oxidant present in FBS and has also been observed that the anti-oxidant play important role in the maturation of oocytes as they are the scavenger of free radicals. It can thus be concluded that FBS is better than EBS for *IVM* of buffalo oocytes, although EBS might be used as serum substitute for maturation of oocytes but rate was slower instead of FBS.

## Authors’ Contributions

GP along with SSC designed the experiment and conducted the experiment with the help of AKS and VKS. GP analyzing the data and preparing the manuscript. GP, SSC, VKS and AKS reviewed the manuscript. All authors read and approved the final manuscript
